# The quest for GAS vaccine

**DOI:** 10.18632/oncotarget.6140

**Published:** 2015-10-16

**Authors:** Manisha Pandey, Michael F. Good

**Affiliations:** Institute for Glycomics, Griffith University, Gold Coast, Australia

**Keywords:** GAS vaccine, memory B cells, combination vaccine, skin infection, invasive infection

The pursuit of a safe vaccine against Streptococcus *pyogenes* (group A streptococcus, GAS) that does not induce autoimmune pathology and that affords coverage for most GAS serotypes, has been a major goal for several decades. These infections account for over 500,000 premature deaths every year resulting from deep tissue infections and post-streptococcal autoimmune sequelae. Despite substantial concerns regarding the safety and efficacy of candidate vaccines, significant advancement has been accomplished. However, a vaccine is still not available, but leading candidates are under development.

Individuals living in GAS-endemic regions or even in high-density areas, are exposed to a number of circulating GAS strains. The ever-changing strain diversity in tropical settings is reported to be significantly high compared to industrialized nations (reviewed in Smeesters et al., 2009 [1]). Evidence suggests that exposure to GAS results in type-specific immunity that protects against the same serotype but not against heterologous strains. While it might be expected that repeated exposure to GAS would result in antibodies to conserved determinants of the M protein, this is not usually the case. The epitopes are hidden or ‘cryptic’. Nevertheless, cryptic epitopes presented in a different context are often no longer cryptic and can be highly immunogenic. Antibodies induced by cryptic epitopes presented out of context may bind the partially concealed epitope on the native protein. A vaccine encompassing highly conserved epitopes could provide coverage against multiple serotypes. There are thus two main approaches to a GAS vaccine that focus on the M protein–using multiple immune-dominant epitopes or a conserved (cryptic) epitope.

The first approach is to combine multiple epitopes representing serotypic determinants from common strains. However, such a vaccine may be region-specific [1]. It is of great interest that vaccination of rabbits with a 30-valent vaccine generated sera that opsonized vaccine GAS strains but also strains of many *emm*-types that were not included in the vaccine, suggesting a high degree of cross-protection for non vaccine M types [2]. Sanderson-Smith et al. [3] have recently demonstrated that over 220 variants of the M-protein can be functionally classified based on their binding and structural properties into 48 *emm*-clusters which could be responsible for the observed cross-reactivity.

Our approach was different and aimed to identify a conserved protective epitope on the M-protein of GAS leading us to describe a cryptic epitope,“J8” [4]. The major advantage of such an epitope is that it is highly conserved, presumably as it is not under significant immune pressure. J8 conjugated to a carrier molecule, DT, has shown broad-spectrum efficacy against systemic as well as intranasal GAS infections of multiple GAS serotypes [5]. However, studies of GAS epidemiology suggesting an association of GAS skin infection with rheumatic fever/rheumatic heart disease in tropical settings, directed the focus of our research towards a vaccine to prevent pyoderma. The development of a murine superficial skin challenge model that mimics human GAS pyoderma, allowed the assessment of vaccine efficacy against skin infection and septicaemia [6]. Intra-muscular immunization with J8-DT adjuvanted with Alum provided strong protection against skin disease that was independent of the *emm* type of the infecting strain. This murine model provides a platform to extend pre-clinical studies and define correlates of protection, giving it tremendous potential for translation into clinical studies.

However, the longevity of immune responses following vaccination is critical. Even more critical is the ability to respond upon re-exposure to the organism at a time when antibody responses have waned completely. The absence of an immunological memory response would necessitate frequent vaccinations. J8 contains only 12 amino acids from streptococcus and as such it required conjugation to a carrier protein (diphtheria toxoid) to provide T-cell help in order to develop an anti-J8 antibody response in an outbred population. There was thus a concern that since DT is not derived from GAS, memory cells would not respond to a subsequent GAS infection. However, we demonstrated that the T-cell help required for activation of a memory B cell (MBC) response could be provided by naive T-cells at the time of re-exposure [7]. Using adoptive transfer of MBC into naïve mice, it was demonstrated that MBC specific for J8, in the absence of detectable serum J8-specific antibodies and memory T cells, were sufficient to protect against skin challenge infection. The data suggest that long after vaccination, an individual would remain protected against subsequent infection [6].

Another potential concern with conserved region M-protein based vaccines has been the possibility of human plasma proteins binding the C-repeat binding region of the M-protein and blocking antibody binding [8]. This is of significant concern, as this would limit the ability of such antibodies to opsonise GAS in the presence of human plasma. This was tested with J8 antibodies in an experimental set-up where anti-J8 antibodies were admixed with human plasma prior to their incubation with live bacteria. Strong binding of J8 antibodies to the GAS surface was observed. In contrast, when GAS was incubated with human plasma prior to addition of J8-antibodies, a significant reduction in binding to GAS surface was observed. These observations were reassuring and suggested that following vaccination of humans with J8-DT, anti-J8 antibodies will compete with albumin and other plasma binding proteins to bind to the C-repeat region enabling opsonin-mediated killing of GAS.

Over the past two decades, a worldwide resurgence of GAS invasive infection has been documented and is attributed to the emergence of highly virulent strains. In most occurrences this is due to mutations in the CovR/S regulon of GAS. A number of GAS virulence factors are up-regulated due to these mutations, including the IL-8 protease, SpyCEP, whose expression is up to 40 times higher in CovR/S mutants. This results in inhibition of neutrophil ingress to the site of infection leading to dissemination of GAS to deeper tissues. Consistent with neutrophils being critical to J8-DT-mediated immunity, J8-DT was found to have significantly compromised efficacy against CovR/S mutants [6]. However, incorporation of a recombinant fragment of SpyCEP with J8-DT resulted in a highly efficacious vaccine that was effective in neutralising SpyCEP, blocking IL-8 degradation and protecting against hypervirulent mutant organisms. We then defined a minimal B-cell epitope on SpyCEP which demonstrated comparable vaccine activity to recSpyCEP (manuscript submitted).

Taken together, all the available data strengthen the need as well as the potential benefits of a GAS vaccine. A broad-spectrum, minimal epitope vaccine would prevent acute infections and significantly reduce any chance of post-streptococcal autoimmune sequelae.
